# The correlation between radiographic and pathologic grading of lumbar facet joint degeneration

**DOI:** 10.1186/s12880-016-0129-9

**Published:** 2016-03-29

**Authors:** Xin Zhou, Yuan Liu, Song Zhou, Xiao-Xing Fu, Xiao-Long Yu, Chang-Lin Fu, Bin Zhang, Min Dai

**Affiliations:** Department of Orthopedics, The First Affiliated Hospital of Nanchang University, Nanchang, 330006 China; Artificial Joint Engineering and Technology Research Center of Jiangxi Province, Nanchang, 330006 China

**Keywords:** Lumbar facet joint, Degeneration, Radiography, Pathology, Spine non-fusion technique

## Abstract

**Background:**

Before performing spine non-fusion surgery that retains the facet joints, choosing an accurate radiographic method to evaluate the degree of facet joint degeneration is extremely important. Therefore, the objective of this study was to determine the accuracy and reliability of different radiographic classifications by analyzing the correlation between radiographic and pathologic grading of lumbar facet joint degeneration. Taking the pathologic examination as standard, the consistency of computed tomography (CT) and magnetic resonance imaging (MRI) assessment of lumbar facet joint degeneration was compared.

**Methods:**

A total of 74 facet joints obtained from 42 patients who underwent posterior lumbar surgery were evaluated. All patients underwent CT and MRI before surgery. The pathologic grade was evaluated with a method based on hematoxylin-eosin and toluidine blue staining. The radiographic grade was evaluated using the methods proposed by different authors.

**Results:**

There was a moderate consistency between pathologic and radiographic grading for facet joint degeneration. The weighted kappa coefficients comparing pathologic with radiographic grading were 0.506 for CT, 0.561 for MRI, and 0.592 for CT combined with MRI, respectively. Taking the pathologic examination as standard, the consistency of CT and MRI examination was also moderate, and the weighted kappa coefficient was 0.459.

**Conclusion:**

The radiographic examination has moderate accuracy and reliability for evaluating degeneration of facet joints. Therefore, a more accurate method for evaluating the degeneration of facet joints is necessary before performing spine non-fusion surgery that retains the facet joints.

**Electronic supplementary material:**

The online version of this article (doi:10.1186/s12880-016-0129-9) contains supplementary material, which is available to authorized users.

## Background

In patients with low back pain, the proportion of lumbar facet joint osteoarthritis (FJOA) is as high as 40–85 % [1]. It has been reported that 15–40 % of low back pain may be caused by FJOA [2, 3]. The facet joint is a synovial joint composed of cartilage, synovium, and an articular capsule [4, 5]. The characteristics of FJOA are similar to other synovial joints such as the knee [4, 6]. The degeneration of the lumbar facet joint will not only cause low back pain but also lead to instability of the spine, resulting in degenerative spondylolisthesis and scoliosis [7].

Spinal fusion is currently the most common operation for treatment of lumbar degenerative disease, but postoperative complications such as loss of motion and adjacent segment degeneration may occur [8]. Motion preservation devices and intervertebral disc replacement may help to reduce these disadvantages. Moreover, interspinous devices have been used to treat low back pain originating from facet joints, but were not suitable for severe facet joint pain [9]. Patients with severe degeneration of the facet joint could still have low back pain after successful intervertebral disc replacement [10]. Therefore, preoperative accurate assessment of facet joint degeneration will contribute to the choice of the appropriate surgical treatment of lumbar degenerative disease.

Recently, the pathologic grading of facet joint degeneration described by Gries [11] has been widely accepted. Radiographic grading was evaluated with methods reported by Pathria, Grogan, and Weishaupt, respectively [12–14]. Determination of the correlation between facet joint pathologic and radiographic grading to facilitate the choice of an appropriate radiographic examination for evaluation of facet joint degeneration is necessary. Our study evaluated the correlation between pathologic and radiographic grading to determine the accuracy and reliability of radiographic grading of facet joint degeneration to facilitate accurate evaluation of facet joint degeneration before lumbar spine surgery, and to aid in selection of the appropriate operation.

## Methods

### Subjects

We recruited 42 patients (19 women and 23 men), 21 to 68 years old (mean: 52 years), who underwent posterior lumbar surgery after being symptomatic for 3 to 240 months (mean: 48 months). All patients underwent CT and MRI before surgery, and 74 inferior articular processes (2 facets at L1/2, 3 at L2/3, 17 at L3/4, 35 at L4/5, and 17 at L5/S1) were obtained at surgery.

We included patients with lumbar spinal stenosis, lumbar disc herniation, and spondylolisthesis. All patients underwent routine CT (64-layer, high-speed helical CT, Siemens) and MRI (1.5 T, Siemens) preoperatively, and the inferior articular processes were resected intraoperatively. Exclusion criteria were patients with lumbar spinal tumor, infectious disease, fracture, or prior surgical treatment.

### Image evaluation

**Fig. 1:**
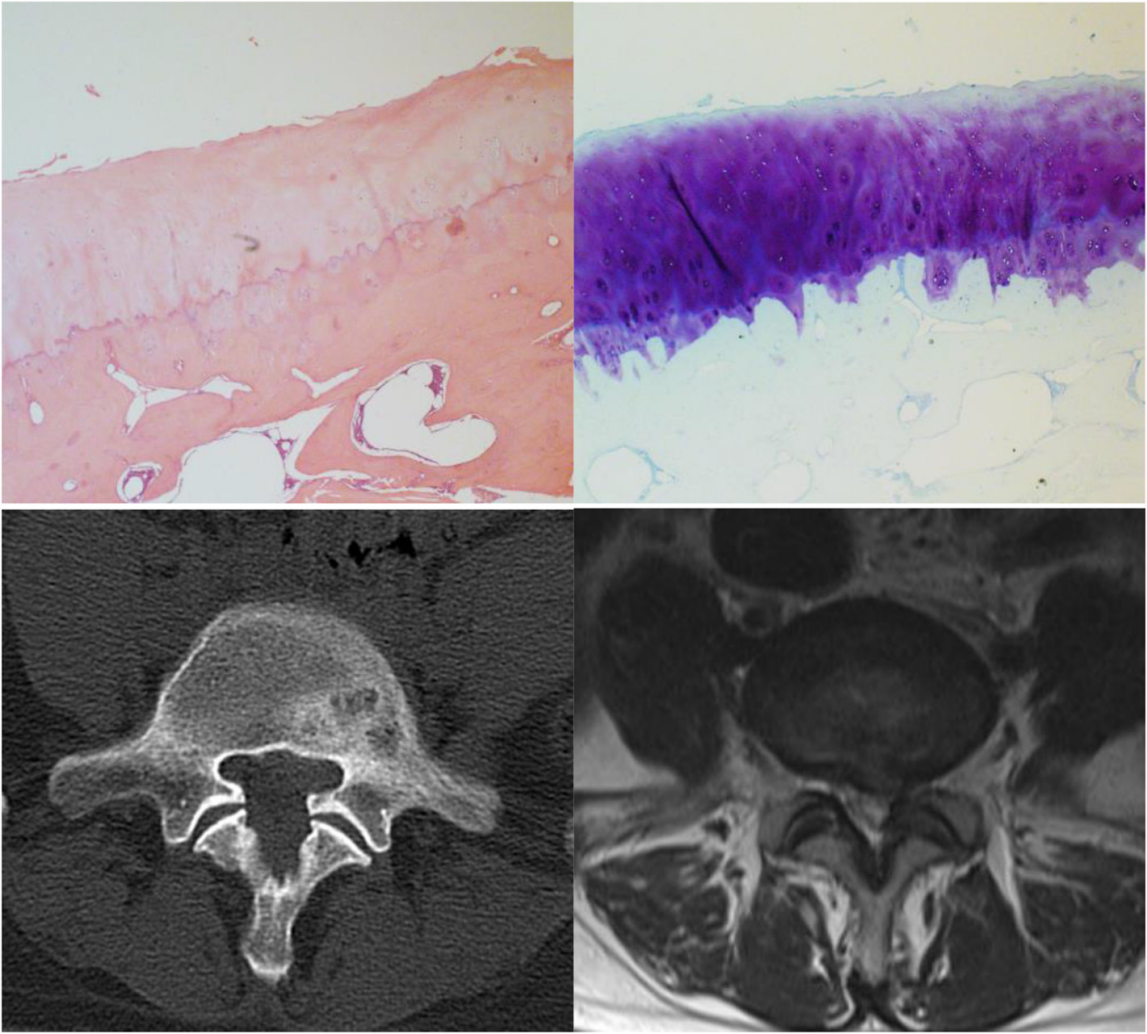
A 43 years old woman with L5-S1 lumbar disc herniation suffered posterior lumbar surgery. The left inferior articular process of L5 was examinated by hematoxylin and eosin (40×) (left) and toluidine blue (40×) (right) stain. The pathologic grading was 2. The CT grading, the MR grading and the CT combined MR grading were also 2

**Fig. 2:**
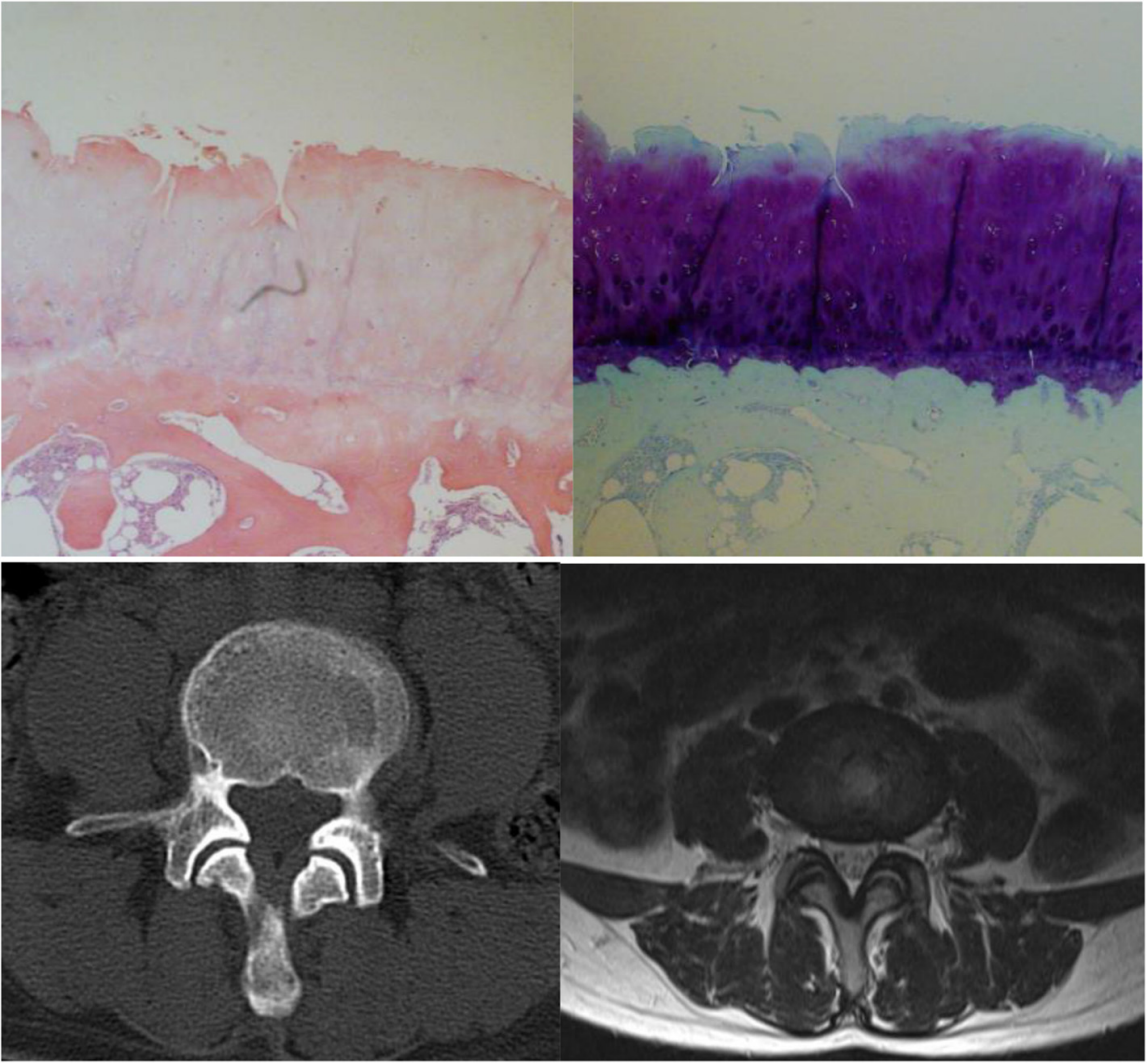
The pathologic grading of the left L4/5 facet joint was 3. While the CT and MR grading was 2, the CT combined MR grading was 3. Both the CT and the MR grading underestimated the degree of facet joints degeneration

**Fig. 3:**
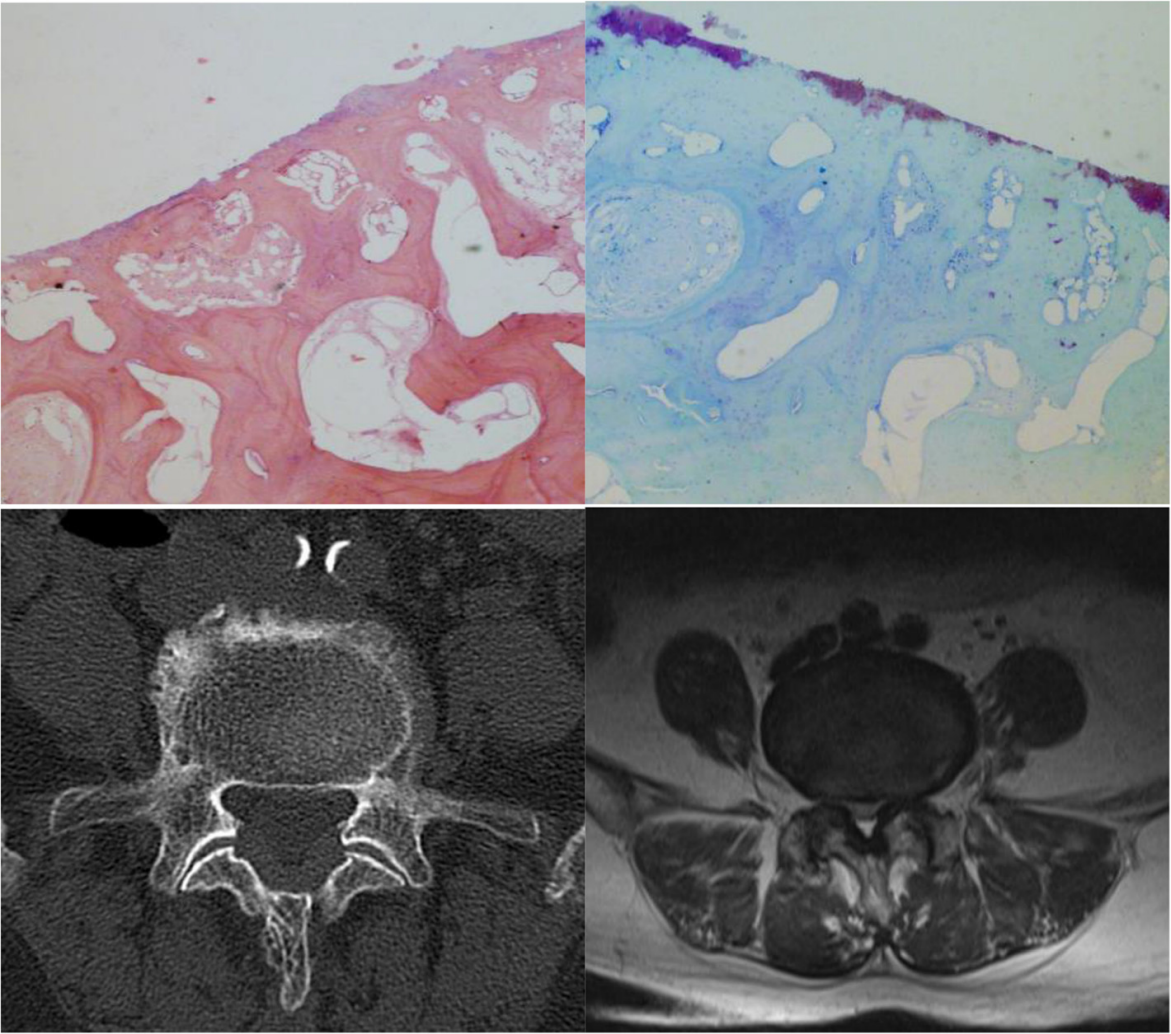
The pathologic grading of the right L4/5 facet joint was 4. While the CT grading was 3, the MR and CT combined MR grading was 4. The CT grading underestimated the degree of facet joints degeneration

Criteria proposed by Pathria to estimate the degeneration of the facet joint on CT were used [12]. Grade 1, normal; Grade 2, narrowing of facet joint; Grade 3, narrowing plus sclerosis or hypertrophy; and Grade 4, severe osteoarthritis with narrowing, sclerosis, and osteophytes (Figs. 1, 2 and 3).

Degeneration of the facet joint on MRI was evaluated according to the criteria used by Grogan [13]. Grade 1, uniformly thick cartilage covering both articular surfaces completely; a uniform thin band of cortical bone. Grade 2, cartilage covering the entire surface with eroded or irregular regions; a thin band of cortical bone extended into the space from the articular surface. Grade 3, cartilage incompletely covering the articular surface, with the underlying bone exposed to the joint space; dense bone extended into the joint space but covering less than half the facet. Grade 4, complete absence of cartilage except for traces evident on the articular surface; presence of osteophytes or dense cortical bone covered greater than half the facet joint (Figs. 1, 2 and 3).

We also used Weishaupt proposed criteria adapted from those by Pathria to define the degree of facet degeneration using CT combined with MRI [14]. Grade 1, normal facet joint space (2–4 mm width); Grade 2, narrowing of the facet joint space (<2 mm) and/or small osteophytes, and/or mild hypertrophy of the articular process; Grade 3, narrowing of the facet joint space and/or moderate osteophytes, and/or moderate hypertrophy of the articular process, and/or mild subarticular bone erosions; and Grade 4, narrowing of the facet joint space and/or large osteophytes, and/or severe hypertrophy of the articular process, and/or severe subarticular bone erosions, and/or subchondral cysts (Figs. 1, 2 and 3).

### Pathologic evaluation

The inferior articular processes were resected during the posterior lumbar surgery, and were fixed in 10 % neutral buffered formalin. The specimens were immersed in a solution containing 10 % nitric acid and 1 % ethylenediaminetetraacetic acid (EDTA) for decalcification. After dehydration the specimens were embedded in paraffin, and a microtome was used to section the specimens into 5-um thickness, followed by dewaxing and washing. Finally the sections were stained by hematoxylin-eosin and toluidine blue, respectively.

The pathologic grade was evaluated with a method proposed by Gries [11]. Grade 1, smooth intact surface, orderly chondrocyte distribution, orderly collagen framework; uniform lamellar subchondral bone plate, uniform vascular budding into cartilage. Grade 2, tangential surface flaking, minimal chondrocyte death, few chondrones; minor thickening of trabeculae, small fissures at bone-cartilage junction, occasional fibrous tissue formation. Grade 3, fissures < 1/2 total depth, loss of cartilage < 1/2 depth, moderate chondrocyte death, many chondrones; moderate trabecular thickening, woven bone formation, moderate fibrous tissue formation. Grade 4, deep fissures, areas of total cartilage loss, extensive chondrocyte death; eburnation of exposed bone, bone sclerosis, cysts, extensive fibrosis (Figs. 1, 2 and 3).

### Statistical analysis

The consistency of radiographic and pathologic grading as well as the consistency of CT and MRI grading based on the histologic examination were evaluated by consistent percentage and weighted kappa statistics. The kappa scores were classified into six categories: less than 0.00 (poor), 0.00 to 0.20 (slight), 0.21 to 0.40 (fair), 0.41 to 0.60 (moderate), 0.61 to 0.80 (substantial), and 0.81 to 1.00 (almost perfect) [15].

All radiographic and pathologic grading was assessed by two independent professionals. Grading was reevaluated up to 4 weeks after the first assessment. The interobserver and intraobserver agreement was estimated. The sensitivity, specificity, false negative rates (FNR).and false positive rate (FPR) were also calculated. SPSS (SPSS Statistics 13) was used for the statistical analyses.

## Results

### Consistency of radiographic and pathologic grading

The results showed moderate consistency between the CT and pathologic grading. Results for readers 1 and 2 and the consensus evaluation were the same for image and histologic grading in 39 (52.70 %), 41 (55.41 %), and 51 (68.92 %) of 74 facets, respectively. The agreement of CT and pathologic grading showed weighted kappa coefficients of 0.291, 0.297, and 0.506 for readers 1 and 2 and the consensus evaluation, respectively. Readers 1 and 2 and the consensus evaluation underestimated 26 (35.14 %), 23 (31.08 %), and 16 (21.62 %) facets (rate), and overestimated 9 (12.16 %), 10 (13.51 %), and 7 (9.46 %) facets (rate), respectively (Table 1). With the pathologic grade set as a standard, and with pathologic grades 1 and 2 defined as not degeneration, and grades 3 and 4 defined as degeneration, the facets were graded as not degeneration by CT, but as degeneration by pathologic grading in 19 (25.68 %), 14 (18.92 %), and 8 (10.81 %) facets by readers 1 and 2 and the consensus evaluation, respectively. The false negative rate (FNR) was 31.67, 23.33, and 13.33 %, and the false positive rate (FPR) was 28.57, 42.86, and 21.43 % for readers 1 and 2 and the consensus evaluation. The sensitivity and specificity of CT were 68.33, 76.67, and 86.67 %, and 71.43, 57.14, and 78.57 % for readers 1 and 2 and the consensus evaluation, respectively (Table 4).

**Table 1:** Consistency of CT grading and pathologic grading for facet joint degeneration

Reader	Underestimate	Exact estimate	Overestimate	Weighted kappa coefficient
Reader 1	26(35.14 %)	39(52.70 %)	9(12.16 %)	0.291
Reader 2	23(31.08 %)	41(55.41 %)	10(13.51 %)	0.297
Consensus	16(21.62 %)	51(68.92 %)	7(9.46 %)	0.506

The results showed moderate consistency between MRI and pathologic grading. Results for readers 1 and 2 and the consensus evaluation were the same grade for images and histologic grade in 49 (66.22 %), 49 (66.22 %), and 54 (72.97 %) of 74 facets, respectively. The agreement of MRI and pathologic grading showed weighted kappa coefficients of 0.458, 0.445, and 0.561 for readers 1 and 2 and the consensus evaluation, respectively. Readers 1 and 2 and the consensus evaluation underestimated 13 (17.57 %), 16 (21.62 %), and 12 (16.22 %) facets (rate), and overestimated 12 (16.22 %), 9 (12.16 %), and 8 (10.81 %) facets (rate), respectively (Table 2). The facets were graded as not degeneration by MRI but as degeneration by pathologic grading in 9 (15 %), 7 (11.67 %), and 6 (10 %) facets by readers 1 and 2 and the consensus evaluation, respectively. The false negative rate was 15, 11.67, and 10 %, and the false positive rate was 50, 42.86, and 35.71 % for readers 1 and 2 and the consensus evaluation, respectively. The sensitivity and specificity of MRI were 85, 88.33, and 90 %, and 50, 57.14, and 64.29 % for readers 1 and 2 and the consensus evaluation, respectively (Table 4).

**Table 2:** Consistency of MR grading and pathologic grading for facet joint degeneration

Reader	Underestimate	Exact estimate	Overestimate	Weighted kappa coefficient
Reader 1	13(17.57 %)	49(66.22 %)	12(16.22 %)	0.458
Reader 2	16(21.62 %)	49(66.22 %)	9(12.16 %)	0.445
Consensus	12(16.22 %)	54(72.97 %)	8(10.81 %)	0.561

The results showed moderate consistency between CT combined with MRI grading and pathologic grading. Results for readers 1 and 2 and the consensus evaluation were the same for images and histologic grade in 45 (60.81 %), 48 (64.86 %), and 55 (74.32 %) of 74 facets, respectively. The agreement of CT combined with MRI and pathologic grading showed weighted kappa coefficients of 0.394, 0.426, and 0.592 for readers 1 and 2 and the consensus evaluation, respectively. Readers 1 and 2 and the consensus evaluation underestimated 23 (31.08 %), 20 (27.03 %), and 14 (18.92 %) facets (rate), and overestimated 6 (8.11 %), 6 (8.11 %), and 5 (6.76 %) facets (rate), respectively (Table 3). The facets were graded as not degeneration by CT combined with MRI but as degeneration by pathologic grading in 15 (25 %), 9 (15 %), and 9 (15 %) facets by readers 1 and 2 and the consensus evaluation. The false negative rate was 25, 15, and 15 %, and the false positive rate was 28.57, 35.71, and 28.57 % for readers 1 and 2 and the consensus evaluation. The sensitivity and specificity of CT combined with MRI were 75, 85, and 85 %, and 71.43, 64.29, and 71.43 % for readers 1 and 2 and the consensus evaluation, respectively (Table 4).

**Table 3:** Consistency of CT combined with MR grading and pathologic grading for facet joint degeneration

Reader	Underestimate	Exact estimate	Overestimate	Weighted kappa coefficient
Reader 1	13(17.57 %)	45(60.81 %)	12(16.22 %)	0.394
Reader 2	16(21.62 %)	48(64.86 %)	9(12.16 %)	0.426
Consensus	12(16.22 %)	55(74.32 %)	8(10.81 %)	0.592

**Table 4:** Sensitivity, specificity, false negative rate, false positive rate of radiographic examination for facet joint degeneration by the pathologic grading

Radiography	Sensitivity	Specificity	FNR	FPR
CT				
Reader 1	68.33 %	71.43 %	31.67 %	28.57 %
Reader 2	76.67 %	57.14 %	23.33 %	42.86 %
Consensus	86.67 %	78.57 %	13.33 %	21.43 %
MR				
Reader 1	85.00 %	50.00 %	15.00 %	50.00 %
Reader 2	88.33 %	57.14 %	11.67 %	42.86 %
Consensus	90.00 %	64.29 %	10.00 %	35.71 %
CT combined with MR				
Reader 1	75.00 %	71.43 %	25.00 %	28.57 %
Reader 2	85.00 %	64.29 %	15.00 %	35.71 %
Consensus	85.00 %	71.43 %	15.00 %	28.57 %

### Consistency of CT and MRI classification based on pathologic grading

With the pathologic grade set as a standard, results for readers 1 and 2 and the consensus evaluation were the same for at least one of the two image grades as for histologic grade in 55, 56, and 63 of 74 facets, respectively. The numbers of CT and MRI classifications which were the same as for the pathologic grade were 38, 41, and 51, and 43, 45, and 54 for readers 1 and 2 and the consensus evaluation, respectively. Results for readers 1 and 2 and the consensus evaluation yielded the same grade for CT and MRI in 26 (47.27 %), 30 (53.57 %), and 42 (66.67 %) facets, and the weighted kappa coefficients were 0.212, 0.235, and 0.459 respectively.

### Intraobserver and interobserver agreement

#### Intraobserver agreement

Two observers evaluated the histologic and radiographic grading twice to determine the intraobserver agreement. The weighted kappa coefficients of the two histology observers were 0.852 and 0.833, respectively (almost perfect).

The weighted kappa coefficients of reader 1 for CT, MRI, and CT combined with MRI grading were 0.655, 0.646, and 0.653, respectively. The weighted kappa coefficients of reader 2 were 0.654, 0.656, and 0.669, respectively; all they corresponded to substantial agreement (Table 5).

**Table 5:** Intraobserver agreement

	Histology		CT		MR		CT combined with MR	
Reader	1	2	1	2	1	2	1	2
Exact estimate	67	66	57	58	57	58	58	60
	90.54 %	89.19 %	77.03 %	78.38 %	77.03 %	78.38 %	78.38 %	81.08 %
Wκ	0.852	0.833	0.655	0.654	0.646	0.656	0.653	0.669

*Wκ* weighted kappa coefficient

#### Interobserver agreement

The weighted kappa coefficients of the two histology observers were 0.810 and 0.812, respectively (almost perfect agreement).

For the first time, the weighted kappa coefficients of readers 1 and 2 for CT, MRI, and CT combined with MRI grading were 0.653, 0.645, and 0.553, respectively. The second time, the weighted kappa coefficients were 0.630, 0.615, and 0.572. The interobserver agreement of the two readers was substantial evaluating facets with CT or MRI, but only moderate combining CT and MRI (Table 6).

**Table 6:** Interobserver agreement

	Histology		CT		MR		CT combined with MR	
Time	1st	2nd	1st	2nd	1st	2nd	1st	2nd
Exact estimate	65	65	57	56	57	56	55	54
	87.84 %	87.84 %	77.03 %	75.68 %	77.03 %	75.68 %	74.32 %	72.97 %
Wκ	0.810	0.812	0.653	0.630	0.645	0.615	0.553	0.572

*Wκ* weighted kappa coefficient

## Discussion

### Consistency between radiographic and pathologic grading

This study showed that radiographic grading of facet joint degeneration demonstrated moderate consistency with pathologic grading; CT combined with MRI grading exhibited the best agreement, followed by MRI grading and CT grading. The sensitivity of evaluation of facet joint degeneration was better than the specificity, indicating that imaging examination could efficiently detect degeneration of the facet joint, but that accuracy needed improvement. Moreover, imaging classification had a tendency to underestimate degeneration compared to pathologic classification; this finding suggests that clinicians should expect more severe facet degeneration than the degeneration estimated through CT or MRI.

In this study, we adopted the grading system proposed by Grogan, and first evaluated both cartilage and subchondral bone degeneration. Studies had shown that subchondral bone plays an important role in the development of osteoarthritis [16–19]. In the early period of osteoarthritis, transformation enhancement of subchondral bone, change of trabecular bone structure, and sclerosis of subchondral bone appear [20]. Because articular cartilage derives nutrition from the terminal vessels in the subchondral bone plate and calcified cartilage layer [21], subchondral bone sclerosis can not only accelerate the disease process, but also is likely to be an originating factor in the onset of osteoarthritis [22]. Considering the role of the subchondral bone in osteoarthritis, this study introduced the grade of facet joint subchondral bone degeneration to obtain more accurate radiographic and pathologic classification.

Since CT examination could better display osteophyte formation, hypertrophy of articular processes, sclerosis, calcification of the joint capsule, and the vacuum joint phenomenon [23], previous research reported that CT was the best radiographic examination for evaluating facet joint degeneration [4, 24–26]. However, our study found that MRI examination was slightly superior to CT in assessing facet joint degeneration. The different results may be explained because the studies that reported CT facet evaluation being better than MRI assessment were published years ago, when MRI technique was limited, thus leading to low accuracy on MRI examination.

Our study used a 1.5 T MRI, which not only better displayed the articular cartilage, joint fluid, and joint capsule, but also showed osteophytes, subchondral bone, and other osseous structures. Use of a 3 T MRI device may be able to identify minimal and early phase degeneration of facet joints, and improve the accuracy of MRI examination. The intraobserver and interobserver agreements of MRI classification were inferior to CT, indicating that MRI grading was more prone to produce divergence between the observers. Therefore, trained and experienced observers are needed to evaluate MRI grading to obtain adequate accuracy.

In this study, the consistency between CT combined with MRI grading and pathology grading was better, the sensitivity was highest, and the false negative rate was lowest than other method alone. However, both CT and MRI examination underestimated facet joint degeneration; thus, advanced imaging technology and more accurate grading methods for facet joint degeneration are necessary to improve the accuracy and reliability of evaluation of the degenerative facet joint.

### Consistency of CT grading and MRI grading based on pathologic grading

With the pathologic examination set as a standard, the CT and MRI grading showed moderate consistency. This result may be related to the features of CT and MRI examination, in which CT mainly observed the degeneration of bony structures, whereas MRI detected articular cartilage degeneration. Therefore, we do not think that CT examination can replace MRI for evaluation of facet joint degeneration.

### Clinical implications of radiographic grading for facet joint degeneration

Facet joint degeneration is an important cause of low back pain [2–4], and the amount of low back pain is associated with the degree of facet joint degeneration in some patients [27]. In the spine arthrodesis, facet joints are usually fused along with intervertebral fusion. As a result, the possible facet pain may be cured, in other words, the facet pain may be eliminated through the fusion procedure. Nevertheless, in non-fusion surgeries that retain the facet joints, such as artificial disc replacement or discectomy, patients with low back pain may still have symptoms secondary to facet joint degeneration. Therefore, an accurate evaluation of facet joint degeneration is particularly important before surgery. This study found that CT and MRI examination in the evaluation of facet joint degeneration had moderate accuracy and reliability, and CT combined with MRI was the best choice for assessment of facet joint degeneration. Clinically, use of CT and MRI examination to evaluate facet joint degeneration before spinal non-fusion surgery is presently the best option to detect FJOA.

There were some limitations in this study. First, patients with lumbar spinal stenosis, lumbar disc herniation, or spondylolisthesis were chosen in this study, and were not clearly diagnosed with FJOA. Therefore, this study did not evaluate the correlation between symptoms and facet joint degeneration. Second, the majority of patients had a long course of disease and severe degeneration of the facet joints, and normal facet joint specimens were not obtained. Increasing the sample size or collecting facets from patients with lumbar fractures could be implemented in further research. Third, the facet samples were excised from living patients, so only the inferior articular specimens which were resected during fusion surgery were used. However, previous studies proved that the degeneration of superior and inferior articular facets made no obvious difference [28, 29]. Thus, we surmise that the inferior articular processes represent degeneration of the entire facet joint.

Facet joints play an important role in non-fusion surgery, but our study showed that the accuracy and reliability of the radiographic examination to evaluate facet joint degeneration was still limited. Therefore, using more advanced radiographic technology and thin-layer scanning, and developing more accurate and effective radiographic grading for facet joint degeneration will be the direction of our further research.

## Conclusion

This study found that current radiographic techniques had moderate accuracy and reliability for assessing facet joint degeneration. CT combined with MRI was better for assessing facet joint degeneration than CT or MRI alone. However, more accurate radiographic grading for evaluating facet joint degeneration is still needed.

### Ethics and consent statements

This study conformed to human experimentation standards of the ethics committee of the First Affiliated Hospital of Nanchang University, and informed consents were obtained from the subjects.

### Availability of data and materials

The dataset supporting the conclusions of this article is included within the article and its Additional file 1.

## Additional file

Additional file 1: Date set (XLS 33 kb)

## Abbreviations

CT: computed tomography
FJOA: facet joint osteoarthritis
FNR: false negative rate
FPR: false positive rate
MRI: magnetic resonance imaging

## References

[1] Goode AP, Carey TS, Jordan JM (2013). Low back pain and lumbar spine osteoarthritis: how are they related?. Curr Rheumatol Rep.

[2] Schwarzer AC, Aprill C, Derby R (1994). Clinical features of patients with pain stemming from the lumbar zygapophyseal joints. Is the lumbar facet syndrome a clinical entity?. Spine.

[3] Manchkanti L, Pampati V, Fellows B (1999). Prevalence of facet joint pain in chronic low back pain. Pain Physician.

[4] Kalichman L, Hunter DJ (2007). Lumbar facet joint osteoarthritis: a review. Semin Arthritis Rheum.

[5] Fujiwara A, Lim TH, An HS (2000). The effect of disc degeneration and facet joint osteoarthritis on the segmental flexibility of the lumbar spine. Spine.

[6] Gellhorn AC, Katz JN, Suri P (2013). Osteoarthritis of the spine: the facet joints. Nat Rev Rheumatol.

[7] Louis R (1985). Spinal stability as defined by the three-column spine concept. Anat Clin.

[8] Kumar MN, Jacquot F, Hall H (2001). Long-term follow-up of functional outcomes and radiographic changes at adjacent levels following lumbar spine fusion for degenerative disc disease. Eur Spine J.

[9] Mario C, Alexander A, Christian W (2009). The short- and mid-term effect of dynamic interspinous distraction in the treatment of recurrent lumbar facet joint pain. Eur Spine J.

[10] Trouillier H, Kern P, Refior HJ (2006). A prospective morphological study of facet joint integrity following intervertebral disc replacement with the CHARITE Artificial Disc. Eur Spine J.

[11] Gries NC, Berlemann U, Moore RJ (2000). Early histologic changes in lower lumbar discs and facet joints and their correlation. Eur Spine J.

[12] Pathria M, Sartoris DJ (1987). Osteoarthritis of the lumbar facet joints: accuracy of oblique radiographic assessment. Radiology.

[13] Grogan J, Nowicki BH, Schmidt TA (1997). Lumbar facet joint tropism does not accelerate degeneration of the facet joints. Am J Neuroradiol.

[14] Weishaupt D, Zanetti M, Boos N (1999). MR imaging and CT in osteoarthritis of the lumbar facet joints. Skeletal Radiol.

[15] Landis JR, Koch GG (1977). The measurement of observer agreement for categorical data. Biometrics.

[16] Sniekers YH, Intema F, Lafeber FP (2008). A role for subchondral bone changes in the process of osteoarthritis: a micro-CT study of two canine models. BMC Musculoskelet Disord.

[17] Botter SM, Van Osch GJ, Waarsing JH (2008). Cartilage damage pattern in relation to subchondral plate thickness in a collagenase-induced model of osteoarthritis. Osteoarthritis Cartilage.

[18] Botter SM, Van Osch GJ, Clockaerts S (2011). Osteoarthritis induction leads to early and temporal subchondral plate porosity in the tibial plateau of mice: an in vivo microfocal computer tomography study. Arthritis Rheum.

[19] Hayami T, Pickarski M, Zhuo Y (2006). Characterization of articular cartilage and subchondral bone changes in the rat anterior cruciate ligament transaction and meniscectomized models of osteoarthritis. Bone.

[20] Tomoya M, Hiroshi H, Toru O (2007). Role of subchondral bone in osteoarthritis development: a comparative study of two strains of guinea pigs with and without spontaneously occurring osteoarthritis. Arthritis Rheum.

[21] Lyons TJ, McClure SF, Stoddart RW (2006). The normal human chondro-osseous junctional region: evidence for contact of uncalcified cartilage with subchondral bone and marrow spaces. BMC Musculoskelet Disord.

[22] Burr DB (2004). Anatomy and physiology of the mineralized tissues: role in the pathogenesis of osteoarthrosis. Osteoarthritis Cartilage.

[23] Carrera GF, Haughton VM, Syvertsen A (1980). Computed tomography of the facet joints. Radiology.

[24] Raskin SP (1981). Degenerative changes of the lumbar spine: assessment by computed tomography. Orthopedics.

[25] Grenier N, Kressel HY, Schiebler ML (1987). Normal and degenerative posterior spinal structures: MR imaging. Radiology.

[26] Modic MT, Ross JS (1991). Magnetic resonance imaging in the evaluation of low back pain. Orthop Clin North Am.

[27] Suri P, Hunter DJ, Rainville J (2013). Presence and extent of severe facet joint osteoarthritis are associated with back pain in older adults. Osteoarthritis Cartilage.

[28] Tanno I, Murakami G, Oguma H (2004). Morphometry of the lumbar zygapophyseal facet capsule and cartilage with special reference to degenerative osteoarthritic changes: an anatomical study using fresh cadavers of elderly Japanese and Korean subjects. J Orthop Sci.

[29] Tischer T, Aktas T, Milz S (2006). Detailed pathological changes of human lumbar facet joints L1-L5 in elderly individuals. Eur Spine J.

